# Treatment of Peripheral Artery Disease Using Injectable Biomaterials and Drug-Coated Balloons: Safety and Efficacy Perspective

**DOI:** 10.3390/pharmaceutics15071813

**Published:** 2023-06-25

**Authors:** Safi Ur Rehman Qamar, Lemana Spahić, Leo Benolić, Marko Zivanovic, Nenad Filipović

**Affiliations:** 1 Bioengineering Research and Development Centre (BioIRC), Prvoslava Stojanovića 6, 34000 Kragujevac, Serbia; lemana@uni.kg.ac.rs (L.S.); leo.benolic@gmail.com (L.B.); fica@kg.ac.rs (N.F.); 2 Faculty of Engineering, University of Kragujevac, Sestre Janjić 6, 34000 Kragujevac, Serbia; 3 Institute for Information Technologies Kragujevac, University of Kragujevac, Jovana Cvijića бб, 34000 Kragujevac, Serbia; zivanovicmkg@gmail.com

**Keywords:** PAD, nanomaterials, injectable biomaterials, cardiovascular health, cardiology, nanotechnology

## Abstract

The possibility of injectable biomaterials being used in the therapy of peripheral artery disease (PAD) is investigated in this article. We conducted a thorough review of the literature on the use and efficacy of biomaterials (BMs) and drug-coated balloons (DCBs). These BMs included hydrogels, collagen scaffolds, and nanoparticles. These BMs could be used alone or in combination with growth factors, stem cells, or gene therapy. The treatment of peripheral artery disease with DCBs is increasingly common in the field of interventional angiology. Studies have been carried out to examine the effectiveness of paclitaxel-coated balloons such as Paccocath^TM^ in lowering the frequency with which further revascularization operations are required. PCB angioplasty and angioplasty without paclitaxel did not significantly vary in terms of mortality, according to the findings of a recent meta-analysis that included the results of four randomized controlled studies. On the other hand, age was found to be a factor that predicted mortality. There was a correlation between the routine utilization of scoring balloon angioplasty along with DCBs and improved clinical outcomes in de novo lesions. In both preclinical and clinical testing, the Selution^TM^ DCB has demonstrated efficacy and safety, but further research is required to determine whether or not it is effective and safe over the long term. In addition, we reviewed the difficulties involved in bringing injectable BMs-based medicines to clinical trials, including the approval processes required by regulatory bodies. Injectable BMs have a significant amount of therapeutic promise for PAD, which highlights the need for more research and clinical studies to be conducted in this field. In conclusion, this research focuses on the potential of injectable BMs and DCBs in the treatment of PAD as well as the hurdles that must be overcome in order to translate these treatments into clinical trials. In this particular field, there is a demand for further research as well as clinical trials.

## 1. Introduction

Cardiovascular disease (CVD) is the prime cause of fatality worldwide. In 2019, the root cause of 32% of all deaths was CVD, representing a 42% rise since 1990 [1]. CVD is categorized into two types, (1) myocardial infarction (MI) and (2) peripheral artery disease (PAD). PAD is characterized by the tightening of the arteries supplying the lower extremities. Common symptoms of PAD include walking limitations and severe pain. However, in several patients, it may progress to tissue loss and ischemic rest pain [2]. Epidemiological studies revealed that about 200 million people are suffering from PAD worldwide. Among those people, a more significant number include elderly people [3]. In addition, it has been seen that the occurrence of PAD rises with age, obesity, and diabetes. It has been noted that the prevalence of PAD was 20% in individuals over 65 years old [4]. However, the current operative care used for PAD is endovascular revascularization. Furthermore, due to high operative risks, 40% of the patients are not eligible for this treatment [5]. Various prescriptions are often recommended for treating PAD, yet these medications have very low or no effect on the amputation rate and increase in functionality of the limbs [6]. Therefore, an alternative medical approach should be opted to conquer the limitations of the existing approach for PAD. In the past two decades, BMs have appeared as a potential therapeutic approach to fill the current gap in treating PAD. For this purpose, various BMs are now being tested in animals and in vitro environments. Multiple studies highlighted the approach of using injectable BMs, nanoparticle-coated balloons [7], BMs-encapsulated drug delivery catheters [8], and nanoparticle-encapsulated drug-coated balloons [9] are being utilized and tested in in vivo and in vitro environments. To demonstrate platelet-derived nanomotor-coated balloon efficacy in treating atherosclerosis, Huang et al. implanted paclitaxel-coated balloons and nanomotor-coated balloons into the carotid arteries of rabbits. On days 0 and 1, and again on day 28, thermal images were taken. The nanomotor group saw much larger increases in carotid artery temperature in response to NIR irradiation than the paclitaxel group. This demonstrated the nanomotor’s promoting effect on drug retention in the plaque region. Furthermore, it is demonstrated that NIR laser irradiation causes a greater quantity of materials to be present in the blood artery. The nanomotor’s ability to enter the vasculature is facilitated by the NIR laser for the reasons listed below. After 28 days, the artery was dissected in order to examine its congestion state and any signs of injury. The region of hyperplasia in the vessels treated with nanomotors is substantially lower than that treated with paclitaxel, and the blood vessels themselves appear undamaged by the photothermal treatment [7]. Wang et al. compared the in vitro activity of a newly designed paclitaxel-coated balloon with pectin as the excipient to the commercially available shellac balloon and characterized the novel nanoparticle paclitaxel-coated balloon with peptide (Arg-Gly-Asp-Ser, RGDS) derivative RGDS-OC8H17. In vitro investigations revealed that the pectin-RGDS-OC8H17-paclitaxel-coated balloon was smoother and more uniform than the commercially available balloon. Paclitaxel’s low concentration highlighted this disparity. In the in vitro testing, the pectin-RGDS-OC8H17-paclitaxel model revealed an adjustable drug-releasing curve: more than 90% was released in 2 h at 300 rpm and more than 99% was released in 10 min at 1200 rpm. The pectin-RGDS-OC8H17-paclitaxel coating released medication faster than commercial shellac excipient products. However, the authors suggested that this innovative drug-coated balloon must be tested in vivo to prove its clinical relevance [8]. Similarly, Yamamoto et al. used NPs-mediated drug delivery technology and polydimethylsiloxane (PDMS) as a new excipient to make it easier for endothelial cells to take in the drug and deliver it to where it needs to go. PDMS was used to coat the new DCB with 5.6 mg of NPs that contained paclitaxel. By using quantitative coronary angiography (QCA) and optical coherence tomography (OCT), the effectiveness of the new DCB was tested in the rabbit iliac stent model (n = 12) and the pig in-stent restenosis model (n = 8). At 28 days of follow-up in the swine in-stent restenosis model, the area of stenosis was significantly lower in the DCB group than in the control group (0.31 ± 0.05 vs. 0.49 ± 0.06, *p* = 0.04). However, in the rabbit iliac stent model, neither QCA nor OCT analysis showed any significant differences. The results of the study showed that a NPs-coated balloon with paclitaxel and PDMS stopped the growth of smooth muscle cells in a pig model of coronary in-stent restenosis [9]. This review article highlighted the major findings from various studies that were performed using BMs and drug delivery systems, i.e., DCBs for treating PAD.

## 2. Injectable Biomaterials

Previously, growth factors, stem cells, and gene therapy were explored for this treatment of PAD which provided a significant result. Nevertheless, BMs along with these therapies either enhance effective outcomes or act as a standalone treatment for PAD (Table 1). Tang and colleagues introduced human umbilical vein endothelial cells (HUVECs) with hyaluronan (HA) in mice. They observed that HA prolonged and increased the capability of HUVEC to persist and implant into the endothelium and hire host smooth-muscle cells. The mixture of HA + HUVEC increased the angiogenesis and limb perfusion as compared with HA alone or HUVEC alone at a 4-week point in a hindlimb ischemic nude mouse model [10]. Wang and colleagues synthesized collagen scaffold (CS) and embedded them onto bone marrow-derived mesenchymal stem cells (BMSCs) to make a CS-BMSCs conjugate for injecting into the hindlimb ischemic rabbit model. They used the oxygen saturation method as an endpoint of increased angiogenesis and hindlimb perfusion. Their cell-material conjugate boosted angiogenesis and increased hindlimb perfusion after eight weeks of treatment. In addition, they observed that collagen alone also enhanced the capillary density. While on the other hand, the CS-BMSCs conjugate significantly increases oxygen saturation and increased vessel density contrasted with saline, material, and cell controls [11]. Similarly, another group of scientists from Japan utilized a nanoparticle-based system consisting of synthetic hydroxyapatite for the distribution of bone marrow mononuclear cells (BNMCs) into the ischemic limb murine model. They observed improved astereognosis, cell survival and angiogenesis after 7 days of treatment. They associated their findings with increased levels of vascular endothelial growth factor (VEGF) and basic fibroblast growth factor (bFGF) protein in the tissue [12].

**Figure 1:**
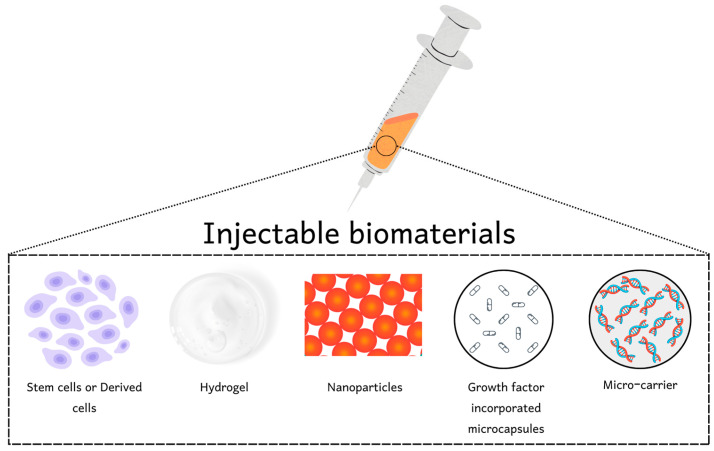
Examples of various types of biomaterials.

Various scientific groups have performed experiments to embed growth factors (EGFs) in material as a technique to enhance the sustainable delivery of an angiogenic factor without a difficulty of employing stem cells. Gelatin microspheres are being used in clinical trials to deliver bFGF to ischemic tissues as a sustainable release system [13]. In another study, bFGF was delivered to ischemic tissue via glutaraldehyde-cross-linked gelatin microspheres. The histopathological results of this study indicated increased microvessel density at the target tissue [14] (Figure 1).

Xie et al. performed the controlled delivery of VEGF over 12 days duration using dextran-co-gelatin nanoparticles in an ischemic rabbit model. They reported increased tubular structures, higher capillary density, blood perfusion, and collateral artery formation in the VEGF-NPs-treated group compared to the NPs and no treatment groups [15]. Kim et al. synthesized neuropeptide substance P-coupled with peptide nanofiber to treat PAD in an ischemic mouse model. They reported on the recruitment of mesenchymal stem cells (MSCs) at the ischemic tissue. In addition, they reported on the inhibition of fibrosis, promotion of vascularization, and enhanced tissue perfusion [16].

**Figure 2:**
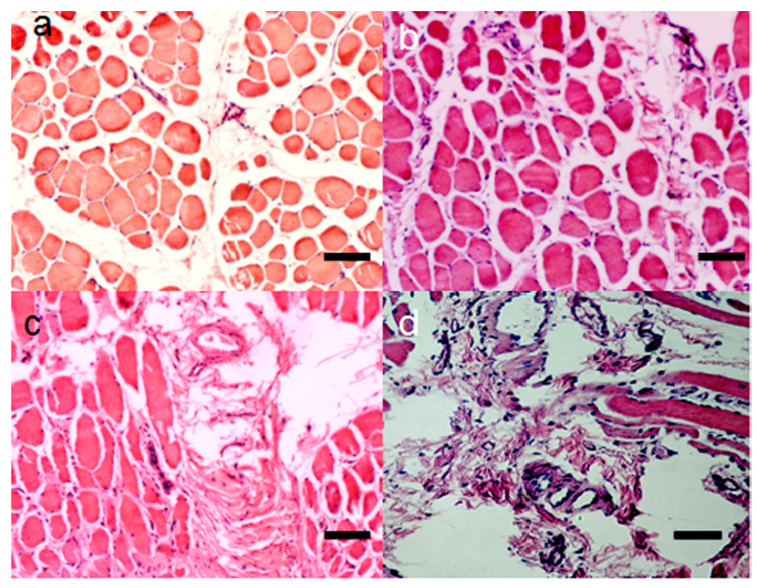
Histological examination: 30 days after implantation, neovascularization in the muscles of the injection location in the (**a**) untreated group, (**b**) empty gelatin microspheres group, (**c**) free basic fibroblast growth factor group, and (**d**) gelatin microspheres–basic fibroblast growth factor group (hematoxylin–eosin, bar = 50 µm). Adapted with permission from [14]. Copyright to publisher Taylor & Francis.

The injection of BMs alone may hold great potential for transformability, as there are limited concerns without extra bioactive components such as EGFs (Figure 2). DeQuach et al. used hydrogel obtained from a decellularized skeletal muscle extracellular matrix. They injected this material for seven days at the target location using an ischemic hindlimb rat model. They reported improved capillary density and arterioles. In addition, the skeletal muscle matrix boosted the employment of MyoD and desmin (+) cells at the ischemia site [17]. Similarly, Fan et al. inserted fibrin (FiB) particles into the ischemic hindlimbs in an ischemic hindlimb rabbit model. Experimental results from calf blood pressure and angiographic score methods reported enhanced arteriole density, capillarization and perfusion after 28 days of treatment [18].

## 3. Challenges in the Application of Injectable Biomaterials

There are presently no FDA-permitted injectable BMs therapies for the treatment of PAD despite encouraging preclinical outcomes. Only two injectable BMs for the heart are presently undertaking clinical trials; both are alginate (Alg) hydrogel systems. One is administered for acute myocardial infraction by trans-coronary catheter infusion, while the other is administered for dilated cardiomyopathy via direct epicardial injection [19,20]. Several clinical studies for growth factor, stem cell, and gene therapy systems have been conducted for PAD, but not one has revealed indisputably beneficial outcomes, and only one of them utilized a BMs scaffold. These scaffolds were the combination of basic fibroblast growth factors (FGFs) and gelatin hydrogel [13]. This inability to translate preclinical findings into clinical research shows that there is a greater need to think of ways to improve the translation of injectable BMs treatments for PAD. Regulatory approval is a significant barrier in the advancement of BMs for cardiovascular disease. Assuming the multiplicity of the BMs treatments, it is challenging to categorize some goods under just one of the FDA’s supervisory branches. The Office of Combination Products (OCP) within the Department of FDA released a draft of a cataloging-related guideline paper to aid in clearing up this misconception [21]. Drugs, biologic and devices items are the three regulatory categories that are highlighted in it. The categorization of a device by the OCP is restricted to any product that does not “achieve its primary planned function through chemical action”. Previously, BMs-based therapies have been essentially categorized as devices under this description. Yet, the OCP emphasizes in the guideline paper that a therapy would not be considered a device if it “depends, even in part, on chemical activity inside or on the body of man to fulfill any one of its major intended functions”. The way the BMs treatment is delivered is another translational issue. Particularly, the way the BMs are administered might influence the simplicity of obtaining regulatory clearance and the probability that hospitals would include the therapy into clinical workflows and procedures. For instance, an injectable myocardial treatment that can be administered during cardiac catheterization processes will likely be simpler to deploy after myocardial infraction than surgically-based epicardial injections. This would eliminate the requirement for common anesthesia and lower the danger to the patient. The majority of injectable substances studied on rats, however, are not responsive to cardiac catheters and the many intracoronary infusions or injections needed [22]. Therefore, it is crucial to take these aspects into account while developing an injectable substance. Moreover, when injectable BMs are employed as part of catheter-based therapies, a link between injectable BMs and cardiac catheterization is formed. To repair damaged or diseased blood arteries, injectable BMs can be employed. After guiding a catheter to a specific region of vascular damage or obstruction, an injectable BM can be administered to the target area via the catheter. Depending on its composition, the BM can then encourage tissue repair, offer mechanical support, or release medicinal chemicals. Therapeutic medications or agents can be delivered directly to the heart or the surrounding vasculature using injectable BMs. Drugs can be released gradually over time by integrating them into the biomaterial matrix, offering long-term therapeutic effects. A cardiac catheter can be utilized to precisely distribute the biomaterial–drug combination into the circulatory system. The use of injectable BMs in conjunction with cardiac catheterization procedures has various advantages over standard surgical treatments. It allows for the targeted and minimally invasive delivery of biomaterials to specific locations of cardiovascular disease, hence lowering the invasiveness and hazards associated with open surgery. Furthermore, because injectable biomaterials can be modified in terms of composition, degradation kinetics, and release patterns, they enable customization and adaptability to particular patient demands.

In addition to the regulatory difficulties connected with a novel BMs treatment, creating and securing FDA certification for a new catheter device will be a burden if a material is incompatible with existing cardiac catheters (e.g., has short gelation durations). Hemocompatibility should also be considered because catheter-based injections are more likely to leak into the systemic flow. These design limitations should be taken into account at the beginning of BMs design and testing to enable faster clinical translation. Finally, PAD employing injectable BMs yields excellent outcomes in terms of both safety and efficacy. Several major conclusions arise from existing research and clinical studies on this subject (see Table 1).

To begin, the safety profile of injectable BMs for PAD treatment looked promising. According to studies, the use of biomaterials such as hydrogels or cell-laden scaffolds is generally well tolerated and does not result in substantial adverse effects or problems (see Table 1). Because these BMs are injectable, they can be used in minimally invasive treatments, which reduces the dangers associated with open surgical techniques. Secondly, the efficacy of injectable BMs in the treatment of PAD appears promising. These materials provide mechanical support, stimulate neovascularization, and can be used to deliver medicinal drugs in a regulated manner. Injectable BMs aid in the restoration of blood flow and tissue oxygenation in damaged limbs by promoting angiogenesis and revascularization. Clinical investigations on PAD patients treated with injectable biomaterials have revealed favorable results in terms of increased walking distance, reduced discomfort, and improved quality of life.

Furthermore, because injectable BMs are versatile and adaptable, individualized and patient-specific therapy techniques are possible. Researchers are investigating the use of diverse biomaterial formulations, such as bioengineered constructions and cell-based therapies, to adapt treatment to the needs of specific patients. This method has a lot of potential for enhancing results and long-term efficacy. Despite the encouraging results, it is critical to recognize that more research and larger-scale clinical studies are required to establish the long-term safety and efficacy of injectable biomaterials for PAD treatment. Furthermore, the ideal biomaterial composition, delivery mechanisms, and treatment regimens necessitate ongoing research and development. In conclusion, the therapy of peripheral artery disease using injectable BMs has a favorable safety profile and promising efficacy in improving blood flow and reducing symptoms. With ongoing advances in biomaterial science and current research initiatives, this technique has the potential to significantly improve the care and outcomes of PAD patients.

## 4. Drug-Coated Balloon

**Figure 3:**
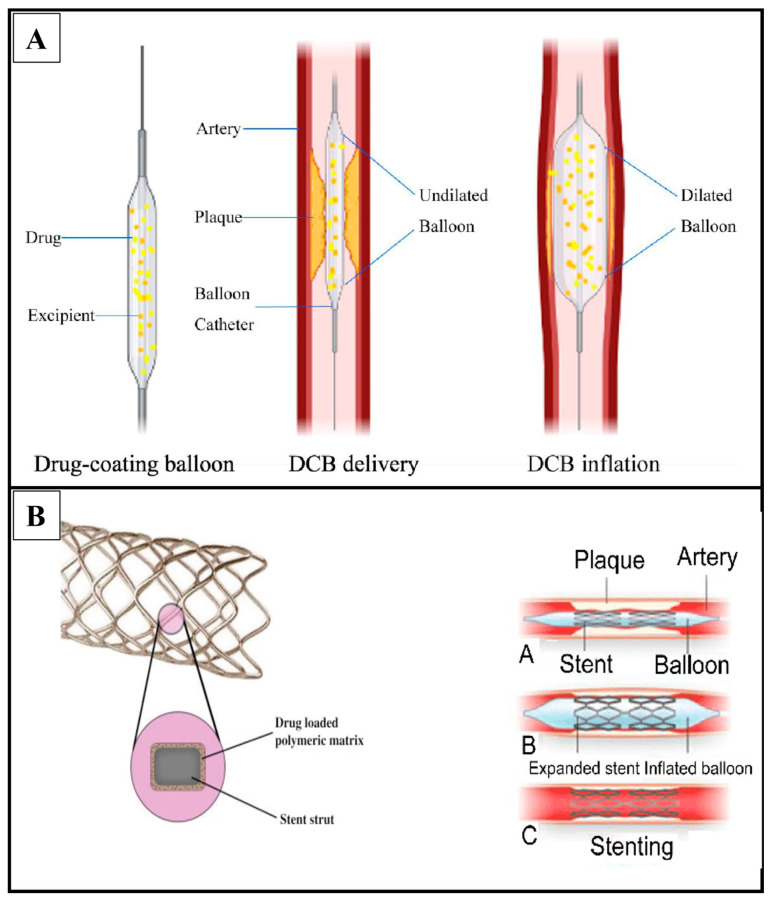
Mechanism of drug release from DCBs and DES. (**A**) In a drug-coated balloon, the drug is put right on the balloon’s surface. When the balloon is put into the target artery and expanded, the drug coating touches the wall of the artery. The drug is then let out of the covering and spreads into the tissue around it. DCBs remove drugs from the body through a process called “transient matrix disruption”. When the balloon is inflated and then deflated, it breaks up the structure of the coating. This lets the drug into the arterial tissue. (**B**) On the other hand, drug-eluting stents are put into an artery to provide artificial support and slowly release the drug. Most stents are made of a metal frame with a polymer covering that has the drug in it. The main ways that drugs are released from DES are through diffusion-controlled release and erosion-controlled release. Adapted with permission from [30,31,32]. Copyright to the publisher Elsevier and Frontiers.

Andreas Grüntzig introduced balloon angioplasty (BA) for the remedy of coronary artery disease in 1977, which was a significant development in the area of cardiology and the beginning of what is now known as contemporary interventional cardiology [23]. In cases when the application of long-term implantation stents is not ideal, drug-coated balloons (DCBs) have become a viable therapeutic option to drug-eluting stents. The idea of administering anti-proliferative medications by balloon angioplasty has been available for a while. The earliest findings is from 1986, when Goldman et al. showed that a multiple lumen polyurethane catheter could effectively deliver horseradish peroxidase (PRXC1A) to human and canine arteries [24]. For the long-term avoidance of restenosis, drug administration and retention are essential. The most effective class of anti-proliferative medications has been observed to be those in the limus family, which are released by last-generation DESs in a regulated manner from an implanted stent [25]. In contrast, DCB releases drugs in a different manner (see Figure 3 for a detailed mechanism).

**Figure 4:**
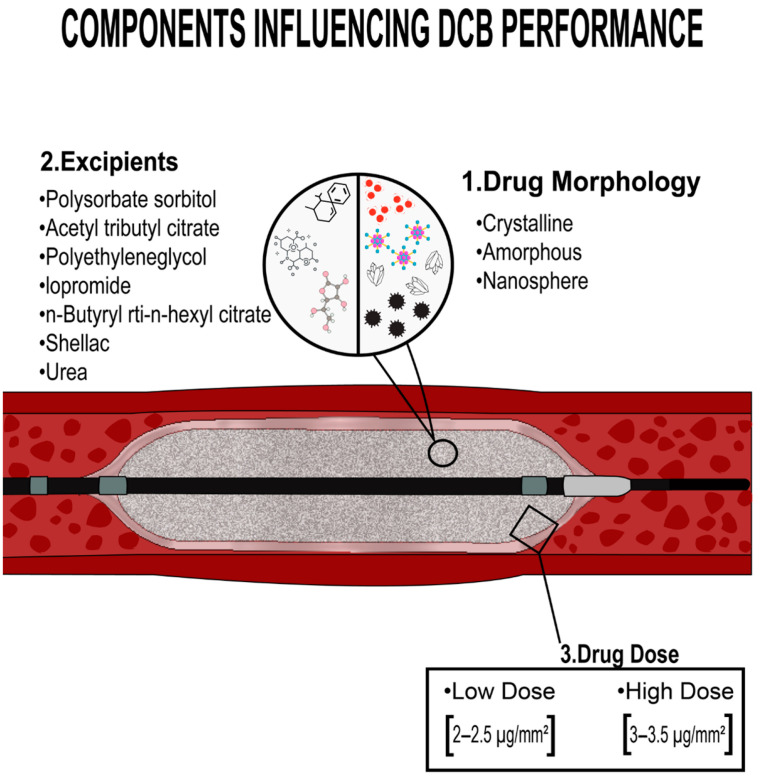
DCB has many features and biological behaviors depending on its shape, excipient, and dosage.

Its effectiveness is dependable on the quick delivery of a particular dose of an anti-proliferative drug, which is anticipated to be incorporated into the vessel wall over time. Edelman et al. explored heparin as one of the first medications for local administration in 1990. They demonstrated that the medication was not kept over time inside the vessel wall as a result of its solubility profile [26]. In 2001, the first agent with favorable outcomes was published by Professor Bruno Scheller. He demonstrated how paclitaxel solubility rose dramatically when coupled with contrast agents, allowing for intracoronary bolus administration and a considerable decline in neointimal proliferation in a pig model of restenosis [27]. The first DCB prototype was created by mixing paclitaxel with contrast media and applying it to the exterior of a balloon [28]. Early clinical evidence stimulated interest in this sector, and other DCBs have since been developed. Programs were developed with the objective of replicating this technical technique by using hydrophilic carriers to transfer paclitaxel through the vessel wall after balloon dilatation. Paclitaxel is used in all commercially marketed DCBs due to its lipophilic nature and chemical stability. Paclitaxel has been shown to suppress cell migration and proliferation by irreversibly alleviating intracellular microtubules, resulting in cell duplication inhibition through metaphase and an anaphase of mitosis [29]. Even though all DCB technologies employ the same basic medication, they differ in a range of ways because of variances in excipients, dosage, production process, and balloon surface technology (Figure 4).

### 4.1. Types of Drug-Coated Balloon

#### 4.1.1. Paclitaxel-Coated Balloons

A. Paccocath^TM^

Paccocath^TM^ paclitaxel balloons use a contrast hydrophilic gap called iopromide as a way to carry the drug. Regular angioplasty balloons with a 3 µg/mm^2^ paclitaxel dosage are known as Paccocath^TM^ balloons. When employed as a matrix, iopromide improves the drug’s release and dissolution, and it may also help the medication attach to the arterial wall. In the initial investigations, paclitaxel and a tiny quantity of the hydrophilic X-ray contrast agent iopromide were used in a pig coronary stent model (Ultravist) to treat neointimal proliferation. Only 12% of the medication was transmitted and 10% was lost in transit in the pig model at a dosage of 2.2 g/mm^2^. Vascular healing in the porcine model was shown to be very modest [33]. The last 20-year literature search showed that very few studies have been carried out using this balloon. In 2019, four randomized controlled trials with clinical data were combined to evaluate 2-year fatalities after paclitaxel-coated balloon (PCB) angioplasty against angioplasty without paclitaxel, i.e., the control group. A PCB coated with a pre-production version of Paccocath^®^ (Cotavance^®^ prototype catheter, Bayer/Medtronic) was utilized in the first two studies (THUNDER and FEMPAC). Medical devices manufactured by Medtronic (InPact^®^ Pacific PCB, Minneapolis, MN, USA) and B. Braun Melsungen AG (SeQuent^®^ Please OTW device, Melsungen, Germany) were used in the PACI-FIER clinical trial. All PCBs were given paclitaxel at a dose of 3.0 µg/mm^2^ along with various excipients. To explore possible predictors of mortality, a logistic regression model was used. After two years, 7.0% (13 out of 185) patients in the control group had died compared to 16/184 (8.7%) in the PBC group, *p* = 0.55. After 2 years, Kaplan–Meier analysis indicated no significant difference from all-cause death (log rank *p* = 0.54). Causes of mortality were evenly distributed between the groups, with no pattern or trend favoring any single cause in the PBC group. Treatment groups (controls or PBC) were not a predictor of 2-year death, according to logistic regression. Patient age above 75 years was the sole predictor of death. During the two-year follow-up period, there was no significant difference in the average daily dose of paclitaxel given to patients who died compared to those who survived (6.248 ± 4.629 μg vs. 5.300 ± 4.224 μg, *p* = 0.433) [34]. In 2020, another clinical study on scoring balloon angioplasty (SBA) was reported by Bonaventura and colleagues [35]. In this prospective, all-comers study (DES-ISR), patients with de novo lesions and in-stent restenotic lesions in bare metal stents (BMS-ISR) and drug-eluting stents (DES-ISR) took part. At 9 months, the main outcome was the rate of target lesion failure (TLF). For the treatment of de novo lesions BMS-ISR (4.0%, 19) (78.4%, 377) and DES-ISR (17.6%, 85), a total of 481 patients (496 lesions) were included. Diabetic mellitus (46.8%, 225), acute coronary syndrome (ACS, 20.6%, 99), and atrial fibrillation (8.5%, 41) were the main risk factors. In the de novo group, average lesion lengths were 16.7 ± 10.4 mm, whereas in the ISR groups, they were 16.2 ± 9.8 mm (DES-ISR) and 20.1 ± 8.9 mm (BMS-ISR). The sizes of the scoring balloons were 2.71 ± 0.31 mm (BMS-ISR), 2.43 ± 0.41 mm (de novo), and 2.92 ± 0.42 mm (DES-ISR), respectively, whereas the diameters of the DCBs were 3.00 ± 0.35 mm (BMS-ISR), 2.60 ± 0.39 mm (de novo), and 3.10 ± 0.43 mm (DES-ISR). Target lesion revascularization rates in the BMS-ISR group (5.3%, 1/19) and the DES-ISR group (6.0%, 5/84) were substantially greater than the total aggregate TLF rate of 3.0% (14/463) in these two groups. The TLF rate for de novo lesions were 1.1% (4/360), and this was not differentiable by calcification status, i.e., non-calcified and calcified lesions (*p* = 0.158) or by reference vessel diameter with a cutoff value of 3.0 mm (*p* = 0.901). High procedural success and low TLF rates have been associated with the routine use of a non-slip element scoring balloon catheter to prepare lesions for drug-coated balloon angioplasty in de novo lesions.

B. NanoPac

A PTCA balloon called NanoPac has paclitaxel on it in a nanocarrier that is enclosed in phospholipid. The medication is contained in a phospholipid bilayer with amphiphilic characteristics with a lipophilic tail and a hydrophilic head. In an in vitro drug release trial, the amount of paclitaxel that was lost during transit was less than 2%, and less than 11% of the drug was still on the exterior of the balloon after the 60 s balloon inflation. The biological effectiveness of a novel lower-dose (2.5 µg/mm^2^) was examined in preclinical research. In a familial hypercholesterolemic swine (FHS) model of iliofemoral ISR, an encapsulated paclitaxel nanocrystal-coated balloon (Nano-PCB) was assessed. In 20 femoral arteries, the pharmacokinetics of nanoPCB were evaluated (of domestic swine). Ten FHS were used to test the biological effectiveness of the procedure. Each stent segment was randomly allocated to receive a clinically available PCB (IN.PACT, n = 14), a Nano-PCB, or an uncoated balloon 14 days after the bare-metal stent was implanted. At 28 days following therapy, an angiographic optical coherence tomography and histological examination were performed. At 1 h, the drug concentration in the artery wall was 120.7 ng/mg, and at 28 days with Nano-PCB, the tissue level was 7.65 ng/mg. In contrast to the baseline, a considerably lower percentage area stenosis was seen (the uncoated specimen and other PCB (Nano-PCB 36.0 ± 14.2%, IN.PACT (Control: 67.9 ± 15.1%, versus 29.3 ± 9.2%; *p* = 0.001)). With the Nano-PCB, the distribution of neointima over the whole stent length was more uniform. Histological analysis indicated that both PCBs had similar levels of neointimal proliferation; however, the Nano-PCB displayed marginally greater stages of neointimal maturity and endothelialization, indicating that the Nano-PCB more effectively promoted endothelialization [36]. More studies needed to be carried out for assessing the actual potential of NanoPac-coated balloons.

C. Pantera Lux

The part of the balloon that goes between the proximal and distal markers of the Pantera Lux is coated with crystalline paclitaxel and a butyryl-trihexyl citrate (BTHC) carrier to help the drug reach the spot of interest. With Pantera Lux coating technology, the folded balloon can have a uniform covering of paclitaxel and the excipient to make the drug more bioavailable. To explore possible vascular impacts of utilizing various excipients, paclitaxel-coated angioplasty balloons were tested in a short preclinical investigation. Four distinct excipients (all 3.0 µg/mm^2^) were used to treat a total of 45 porcine arteries with paclitaxel-coated DEBs: (a) acetyl triethyl citrate (ATEC) excipient (n = 8), (b) iopromide (n = 9), (c) lecithin excipient (n = 10) and (d) BTHC excipient (n = 10).

As controls (n = 8), naked, uncoated angioplasty balloons were used. Histomorphometry, quantitative angiography analysis, and other procedures were performed at 28-day histology. Paclitaxel tissue concentrations were determined in selected rats utilizing the BTHC excipient (n = 39 arteries) with a maximal concentration (C_max_) of 165 ng/mg in the coronary target tissue after 30 min of application. Histomorphology and quantitative angiography efficacy end goals showed no changes across groups. Treatment with DEBs utilizing the BTHC excipient or iopromide, on the other hand, was linked to enhanced fibrin deposition and inflammation, which suggests that vascular repair was delayed. DEBs utilizing uncoated angioplasty balloons or lecithin excipients failed to provide equivalent vascular effects. Up to 7 days after implantation, the paclitaxel concentration in the pig coronary artery was assessed [37]. The existence of variations in clinical results between individuals displaying late or early DES-ISR treated with DCB stays an unanswered scientific question. A literature search provided a detail on a clinical trial carried out in Germany during 2018–2019 using Pantera Lux DCBs [38]. In that study of patients presenting with early (12 months) vs. late (>12 months) DES-ISR who were treated with DCB, clinical outcomes were examined according to the time of incidence of ISR following DES implantation. The study comprised 352 individuals, 199 of whom presented with early-stage ISR and 153 of whom presented with late-stage ISR. Out of the total number of patients (352), 199 showed signs of ISR at an early time point, and 153 showed signs of ISR at a later time point. When comparing patients with early and patients with late DES-ISR, the former group had a significantly greater risk of the main endpoint (25.9% vs. 17.0%; *p* = 0.04). An increased risk for major adverse cardiac events (MACE) was seen between early and late DES-ISR in a multivariate analysis that included diabetes condition, clinical presentation, prior coronary bypass graft, and diameter stenosis after DCB treatment (HRadj = 1.8, [95% CI = 1.1–3.0], *p* = 0.02). Patients presenting with early DES restenosis had a significantly greater clinical event rate than patients presenting with late DES restenosis at 12 months following treatment of DES-ISR with DCB. Recently, another large-scale clinical study aiming to show that Pantera Lux and SeQuent Please DCB therapy is effective and safe for use in a broad, real-world population was published in a Korean circulation journal [39]. In this study, a multicenter observational registry was created for DCB-treated patients in Korea, enrolling patients from 18 institutions between January 2009 and December 2017. TLF was defined as a combination of cardiovascular mortality, myocardial infarction in the target artery, and clinically recommended revascularization of the target lesion within 12 months. In all, 2509 individuals were included in the trial, and a total of 2666 coronary artery lesions were treated with DCBs (1688 (63.3% of total) were in-stent restenosis (ISR) lesions, while 978 (36.7%) were de novo lesions). Patients had a mean age of 65.7 years (SD = 11.3), and 65.7% were male. For the whole population, the ISR group, and the de novo lesion group, TLF occurred in 179 (6.7%), 151 (8.9%), and 28 (2.9%) patients at 12 months. In the whole study population, the independent predictors of 12-month TLF were a history of diabetes, hypertension, prior coronary artery bypass graft, acute coronary syndrome, B2C lesion, decreased left ventricular ejection fraction, and ISR lesion. Patients with ISR lesions and those with minor de novo coronary lesions both benefited clinically from DCB therapy, as was shown in this larger multicenter DCB registry trial. A similar study from Australia was also shown to support the safety of Pantera Lux DCBs. The intention was to evaluate the viability of temporary stenting using a paclitaxel-eluting balloon in the side branch and an everolimus-eluting stent in the main branch. In this prospective investigation, 35 individuals with bifurcation lesions participated in a five-site, multicenter study in Australia. At 9 months, an angiography and intravascular ultrasound were performed, and until 12 months, clinical follow-up was performed. The primary objective was 0.10–0.43 mm, as measured by the quantity of late lumen loss in the side branch. There were no signs of bilateral restenosis. There was one fatality, three myocardial infarctions (two in non-target vessels and one suspected) and one revascularization of a target lesion. No definitive or probable stent thrombosis was observed. Using a combination of an everolimus-eluting stent in the main branch and a paclitaxel-eluting balloon in the side branch, bifurcation lesions can be treated in a safe, effective, and novel manner, according to the findings of the study [40]. A comparative study on the safety of Pantera Lux and SeQuent Please DCBs showed that similar numbers of patients experienced MACE throughout their hospital stay regardless of treatment (Pantera Lux: 6 patients (1.6%) vs. SeQuent^®^ Please: 3 patients (1.5%), relative risk 1.06, 95% confidence range 0.3–4.2, *p* = 0.93). Patients with Pantera Lux DCB had a substantially higher event-free survival rate than those using SeQuent Please DCB (hazard ratio: 0.65, 95% confidence interval: 0.43–0.98; *p*-value of log-rank test: 0.0405) [41]. In conclusion, studies from different countries with different sample sizes indicated that Pantera Lux is a safer approach for treating CAD and PAD. However, all studies have suggested that these findings have to be again confirmed in future clinical studies.

#### 4.1.2. Limus-Coated Balloons

A. MedAlliance Selution^TM^ Sirolimus-Eluting Balloon

The Selution^TM^ DCB makes use of a poly (lactic-co-glycolic) microreservoir. To administer sirolimus at a dosage of 1 mg/mm^2^ with a regulated and sustained drug release mechanism that retains a therapeutic impact in tissue over time, a biodegradable acid polymer (PLGA) was combined with a phospholipid formulation. The microspheres deliver prolonged drug release analogous to that of a DES and are delivered to the vessel wall through balloon inflation. In a preclinical investigation, a chronic restenosis 15% overstretch model in pig coronary arteries was used to assess tissue drug levels and biological response to therapy with the Selution^TM^ DCB. With 36% drug loss and 39% drug transfer to the vessel wall, the Selution^TM^ DCB left 25% of the medication on the balloon. At days 0, 7, 28, and 60, the drug concentrations in tissue were 262, 44, 21, and 19 μg/g, respectively [42]. Scopus and Web of Science searches indicated that not many studies have been published for the assessment of Selution^TM^ DCBs, as we only found total of eight major studies, two of which were clinical design hypothesis for Selution^TM^ DCBs [43,44], and one was case study of two different peoples [45]. The first human trial from Germany (between October 2016 and May 2017) to assess the safety and efficacy of Selution^TM^ DCBs was published in 2020 [46]. After 6 months of Selution^TM^ DCBs therapy, the median angiographic late lumen loss was 0.19 mm (range = 1.16 to 3.07). Compared to the OPC value of 1.04 mm, the mean angiographic late lumen loss (n = 34) was 0.29–0.84 mm (95% CI 0.01 to 0.58). Duplex ultrasonography demonstrated a primary patency rate of 88.4 percent, and angiographic binary restenosis was not seen in 91.2 percent of cases. Through 6 months, both Rutherford categories and ABI values improved significantly from baseline (*p =* 0.001). At 5 months, there was a single occurrence of CD-TLR (2%). No more serious adverse effects occurred. Similarly, another trial was run in Singapore in 2020 (May to November) with a population of n = 39 patients aged 65. The juxta-anastomosis was the most common site of intervention (24/43, or 54.5%), and a decrease in access flow was the most prevalent rationale for intervention. The technical and operational aspects were flawless. There were no SEB-related complications. Primary patency rates in the target lesion were 39/41 (95.1%) at 3 months and 28/39 (71.8%) at 6 months. At 3 months, 35 of 37 (94.60%) access circuits were still functioning, but at 6 months, 22 of 35 (62.91) were still operational. Three fatalities occurred, accounting for 7.7 percent, and they were all caused by pre-existing conditions [47]. A follow-up of this study was published in 2022. At 12 months, there have been no device-related adverse effects following fistuloplasty with the new Selution^TM^ DCBs for defective AVF circuits in Asian hemodialysis patients. Despite promising 6-month performance results, 1-year TLPP and circuit access patency rates are disappointing. This could be because Asian AVFs have more complicated multiple lesions and need more drug to be eluted into the adventitial wall between these two time points to stop the NIH process [48]. Another pilot study from Singapore was carried out by the same research group with a focus on chronic limb-threatening ischemia patients published in 2021. There were 25 patients altogether. There were 17 people (68.0% were men) with a mean age of 63.7 ± 9.73. Based on the Rutherford scale (R5 = 25/25; 100%), CLTI severity was determined. Diabetes mellitus (n = 22; 88.0%) and end-stage renal failure (n = 11; 44.0%) were both significant comorbidities. A total of 33 atherosclerotic lesions (TASC II D = 15 (45.5%)) were treated. The average length of the lesion treated was 191 mm. The technology worked well. Primary tibial patency was 22/27 (81.5%) at 6 months, and 25/30 (83.3%) of patients were free of clinically motivated TLR. Overall, 21/25 (84.0%; three fatalities and one significant lower extremity amputation) were AFS. At six months, the mean Rutherford score increased from 5.00 at baseline to 1.14 ± 2.10 (*p* < 0.05). At three and six months, the wound-healing rate was 13/22 (59.1%) and 17/21 (81.0%), respectively [49]. Later, the follow-up of the same was published in 2022. Through one year, it shows improved cumulative TLR (7.4% vs. 20%), greater AFS (84% vs. 59%), and better wound healing (82% vs. 52%) rates [50]. All these studies provided details that Selution^TM^ DCBs appears to be safe and effective in treating complex tibial arterial occlusive lesions, inhibiting restenosis and dysfunctional AVF circuits in both Asian and German populations. These studies suggested a larger duration trial to confirm the long-term effect of Selution^TM^ DCBs (see Table 2).

B. Magic Touch Sirolimus-Eluting Balloon

A balloon covered with a sirolimus nanocarrier is called Magic Touch (Concept Medical, Surat, India), which was the first balloon with Conformité Européenne (CE) compliance to be deployed on a significant number of patients. The balloon is covered with Nanolute^®^ technology, which delivers sirolimus-containing polymer-free nanocarriers encased in a patented drug carrier called a phospholipid excipient. The FDA has granted the phospholipid excipient drug carrier a GRAS classification. In order to keep sirolimus inside the encapsulation for a longer period of time, the carrier possesses amphiphilic characteristics. The hydrophilic surface of the balloon is covered with nanocarriers in a circumferential pattern. When the balloon has an interaction with the artery lumen, the maximum medication delivery is made possible by the hydrophilic surface of the nanocarrier becoming moist during balloon expansion. Nanocarriers are transported to the artery wall once the balloon is inflated in the arterial lumen to the required nominal pressure (one inflation lasting 60 s or two inflations lasting 30 s each at the target spot). Fick’s equations of diffusion, which say that the difference in the drug’s concentration gradient leads to a homogeneous drug concentration, can be used to explain why the drug moves through the artery wall. With the coating of nanocarriers, there is less drug loss in transit, less particulate matter, better drug diffusion, drug retention throughout the artery, and sirolimus reaches the deep layers of the coronary artery. All of these things lead to greater bioavailability. After a nanocarrier sirolimus-eluting balloon was inflated for 60 s, a pharmacokinetic study was conducted on New Zealand white male rabbits to find out how the amount of sirolimus in their blood and tissues changed over time. The blood levels peaked at 9.3 ng/mL at 30 min after balloon inflation and dropped to 0.8 ng/mL after 24 h. After the index operation, the tissue sirolimus concentration was 140.6 ng/mg on the first day, 15.5 ng/mg after 8 days, and 5.5 ng/mg at 14 days [51]. The excipient dosage was assessed in 14 adolescent domestic pigs weighing between 18 and 23 kg as part of a comparison of the medicines’ safety and effectiveness. After balloon inflation, DTF-labeled sirolimus nanoparticles were examined by confocal microscopy to determine their temporal drug dispersion. A dose-finding assessment study demonstrated progressive neointimal inhibition with the most potent formulation of 1:1. At 1 h, 60–70% of the circumferential area displayed a DTF signal; at 3 days, 30–40% of the circumferential area displayed a DTF signal, with some signals penetrating below the internal elastic lamina and others in the media; and at 7 days, 30–40% of the circumferential area displayed a DTF signal with the presence of a signal in the deep media and a few signals in the adventitia [52]. A registry of 277 patients who had SCB (Magic Touch^TM^ balloon) treatment for both de novo (55.42%) and ISR (44.58%) revealed a major adverse cardiac event (MACE) rate of 5.38% in the 186 patients who have so far undergone 12 months of clinical follow-up. Nevertheless, there are currently no official angiographic follow-up statistics available [53]. Another study was published in 2020 and provided more detail on its efficiency. Caiazzo et al. performed percutaneous coronary intervention (PCI) on 74 consecutive patients, using at least one SCB for ISR and/or de novo coronary lesions in small channels. In their study, the data from 62 individuals who had ACS at the time of presentation were included. The average age of the patients was 67 ± 10 years, and ST-elevated myocardial infarction (STEMI) was found in 14 (23%). ISR was only 48% compared to 52% for de novo lesions. Procedural success was achieved in all instances. Major adverse cardiovascular events (MACEs) were 3 (4.8%) at the 11 ± 7-month follow-up. Ischemia-driven target lesion revascularization and cardiovascular death (CD) occurred in one (1.6%) and two (3.2%) individuals, respectively (TLR). There was one suspected subacute thrombosis (1.6%) but no significant bleeding. Patients treated for de novo lesions and ISR did not vary significantly in terms of MACE incidence in a subgroup analysis (HR: 0.239; CI 95%: 0.003–16.761) [54]. They concluded that it is safe and efficacious for the treatment; however, a larger population study is needed to confirm the findings.

C. Virtue Sirolimus-Eluting Balloon

Caliber Therapeutics Virtue DCB is a microporous angioplasty balloon catheter with a sirolimus nanoparticle formulation. Sirolimus is packed with biodegradable polyester-based polymers in the formulation. The Virtue DCB was tested for practicality, long-term retention, and vascular compatibility. Sirolimus nanoparticles were put into the vessels of regular pigs and a model of ISR pigs. Sirolimus was found in the blood after 1 h (409 ± 78 ng/mL). After 24 h, it was much lower (66 ± 24 ng/mL), and after 96 h, it could not be found at all. In these animals, the highest tissue concentration (C_max_) was 416 ± 77 ng/mL, and it took 11 ± 8 min after the last drug dose for C_max_ to be reached (T_max_). At 28 days, the percentage of stenosis was lower in places where the mixture was used than in places where balloon angioplasty treatment was used. The biggest change was seen in de novo lesions [55]. Through searches from Scopus and Web of Science, we only found one clinical trial. Verheye et al. published only one clinical trial study in 2017. In this study, fifty ISR patients were treated with the Virtue balloon using a single-arm feasibility trial method that took place at nine European locations. The results of the angiographic measures taken at six months and the clinical follow-up at twelve months are presented by them. In the intention-to-treat population, the procedure was completely successful. TLF (cardiac mortality, target vessel myocardial infarction, and clinically induced target lesion revascularization) was the primary safety objective at 30 days (0%, n = 50). After 6 months, the key performance outcome was in-segment late lumen loss (LLL) (0.31 ± 0.52 mm; n = 47). Binary restenosis (19.1%), diameter stenosis (30.3 ± 19.9%), and major adverse cardiac events (MACE) (10.2%, n = 49) are secondary 6-month goals. The LLL was 0.12 ± 0.33 mm at 6 months in the 36-patient per-protocol group (excluding serious protocol violations and previously stented ISR). At one year, the intention-to-treat group had 12.2% TLF and 14.3% MACE, while the per-protocol population had 2.8% TLF and 2.8% MACE [55]. Using the Virtue sirolimus-eluting angioplasty balloon, this first-in-human trial demonstrated outstanding procedural success, 6-month LLL rates consistent with available stent-free ISR therapy options, and clinical outcomes that call for further investigation in specialized randomized studies.

**Table 2:** Summary of studies.

Type of DCB		Drug Excipient	Type of Study	Outcome	Reference
Paclitaxel-Coated Balloons	Paccocath ^TM^	paclitaxel and iopromide	In vivo (pig coronary stent model)	vascular healing in the porcine model	[33]
			Clinical trial (human)	low mortality	[34]
			Clinical trial (human)	high procedural success low TLF rates	[35]
	NanoPac	paclitaxel on phospholipid nanocarrier	In vitro	increased endotheliali-zation more studies needed to be carried out for assessing the actual potential	[36]
	Pantera Lux	crystalline paclitaxel and butyryl-trihexyl citrate	In vivo (porcine arteries) and clinical trials (human)	enhanced fibrin deposition delayed vascular repair increased risk for major adverse cardiac events	[37,38,39,40]
Limus-Coated Balloons	MedAlliance Selution^TM^ Sirolimus-Eluting Balloon	Sirolimus and poly (lactic-co-glycolic) microreservoir	In vivo (pig coronary stent model)	no serious adverse effects effective in treating complex tibial arterial occlusive lesions, inhibiting restenosis and dysfunctional AVF circuits	[43,44,45,46,47,48,49]
	Magic Touch Sirolimus-Eluting Balloon	sirolimus and phospholipid nanocarrier	In vivo (white male rabbits and pigs) and clinical trial (humans)	progressive neointimal inhibition no adverse outcome safe to use in humans	[52,53,54]
	Virtue Sirolimus-Eluting Balloon	sirolimus packed in polyester-based polymers nanoparticle	In vivo (porcine arteries) and clinical trial (human)	reduction in percentage stenosis first-in-human trial demonstrated outstanding procedural success	[55]

## 5. Challenges in the Application of Drug-Coated Balloons

Drug-coated balloons (DCB) were developed as an option to address some of the limitations of previous approaches such as DES. One of the most enticing qualities of these devices is their ability to deliver anti-proliferative drugs into the vessel wall while “leaving nothing behind”. DCBs have a number of potential advantages over DES and other existing treatment methods, including drug administration without the need of a permanent polymer, the capacity to distribute over a larger surface area covered by the balloon, and a shorter duration of dual antiplatelet therapy [56]. Furthermore, DCBs may be used to treat vascular lesions that are not effectively serviced by stents, such as high mechanically stressed areas (tortuous arteries), tiny vessels, widespread lesions, and bifurcation lesions. DCBs deliver uniform and quick anti-proliferative drug delivery to the treated vessel, which inhibits neointimal proliferation. Cost is a significant barrier, and the lack of reliable cost-effective research is a serious problem. Nonetheless, DCBs exhibit the inherent mechanical constraints of balloon angioplasty, most notably a lack of scaffolding and mechanical support, which limits their stand-alone applicability. As a result, if the outcome of the original DCB usage is unsatisfactory (i.e., dissection, recoil, ineffective dilatation), bailout stenting is always required, undermining the aim of its primary prescription. Another possible constraint is the requirement for vascular damage in order to obtain optimal medication distribution. A barotrauma with profound vascular damage is caused by balloon angioplasty. Plaque compression is not a substantial cause of lumen dilation [57], and the main causes of lumen enlargement are plaque rupture with arterial dissection and overstretching of the whole artery wall [58,59]. Additionally, vascular microdissection itself is believed to promote drug transfer and produce appropriate drug bioavailability in the tissue, necessitating a delicate balancing act between the right balloon sizing and degree of vessel damage [60]. These stent-related problems appear to be much greater in aorto-ostial and bifurcation locations, owing to a number of unfavorable rheological, morphological, and histological variables that may be linked with geographic miss, stent malapposition, and/or delayed endothelization [61]. Furthermore, “carina shift” may occur as a serious procedural complication in the therapy of bifurcation stenoses (including distal Left Main Stem Percutaneous Coronary Intervention or LMS), particularly when specific procedures such as cross-over stenting and ostial stenting are used [62].

## 6. Conclusions

Many people suffering from PAD may benefit from acellular biomaterial-based therapy. Patients may soon receive treatments tailored to induce tissue regeneration and enhance muscle function by leveraging the capacity to manufacture these biomaterials and utilize the minimally invasive aspect of many of these therapies. Although more studies, i.e., in vitro and in vivo, are needed to establish appropriate biomaterial techniques as well as manufacturing costs, the area is fast developing toward discovering novel therapies for PAD patients. On the other hand, the paclitaxel balloon is being used in cardiovascular therapy, while the use of a drug-coated balloon to deliver sirolimus is novel. However, in terms of effectiveness, sirolimus is superior. Despite the fact that no standardized pharmacological therapy has been created in the clinical world, bigger randomized clinical studies may produce proof of concept in therapeutic superiority.

## Figures and Tables

**Table 1:** List of various biomaterials with their positive outcome.

Type of Material for Biomaterial Formation	Cell Line Used for Biomaterial Formation	Test Environment	Conclusion	Reference
hyaluronan	human umbilical vein endothelial cells	in vitro (cell lines) in vivo (nude mouse model)	increased angiogenesis. increased limb perfusion	[10]
collagen scaffold	bone marrow-derived mesenchymal stem cells	in vivo (hindlimb ischemic rabbit model)	boosted angiogenesis increased hindlimb perfusion increased oxygen saturation increased vessel density	[11]
NPs system with synthetic hydroxyapatite	bone marrow mononuclear cells	in vivo (ischemic limb murine model)	improved astereognosis increased angiogenesis increased cell survival	[12]
bFGF-incorporated gelatin hydrogel microspheres		clinical trial	improved rest pain scale improved ankle-brachial pressure index improved ulcer healing	[13]
bFGF-incorporated glutaraldehyde-cross-linked gelatin microspheres		in vivo (canine ischemic hind limb)	increased microvessel density	[14]
VEGF-incorporated dextran-co-gelatin NPs		in vivo (ischemic rabbit model)	increased tubular structures higher capillary density increased blood perfusion collateral artery formation	[15]
neuropeptide substance P-coupled with peptide nanofiber		in vivo (mouse hind limb ischemia model)	inhibition of fibrosis promotion of vascularization enhanced tissue perfusion	[16]
hydrogel from decellularized skeletal muscle extracellular matrix		in vivo (ischemic hindlimb rat model)	improved capillary density improved arterioles	[17]
fibrin particles		in vivo (hindlimb ischemic rabbit model)	enhanced arteriole density improved capillarization increased blood perfusion	[18]

## Data Availability

Not applicable.
