# Correction: Qamar et al. Treatment of Peripheral Artery Disease Using Injectable Biomaterials and Drug-Coated Balloons: Safety and Efficacy Perspective. *Pharmaceutics* 2023, *15*, 1813

**DOI:** 10.3390/pharmaceutics15112555

**Published:** 2023-10-30

**Authors:** Safi Ur Rehman Qamar, Lemana Spahić, Leo Benolić, Marko Zivanovic, Nenad Filipović

**Affiliations:** 1 Bioengineering Research and Development Centre (BioIRC), Prvoslava Stojanovića 6, 34000 Kragujevac, Serbia; 2 Faculty of Engineering, University of Kragujevac, Sestre Janjić 6, 34000 Kragujevac, Serbia; 3 Institute for Information Technologies Kragujevac, University of Kragujevac, Jovana Cvijića бб, 34000 Kragujevac, Serbia

There was an error in the original publication [1] comprising a misstatement of the text regarding NanoPac. A correction has been made to:

- Section 4, Subsection 4.1.1, through the removal of “One of the studies has shown that NanoPac is responsible for inducing tumor in an in vivo model [37], while another study has provided the detail on its pharmacokinetics profile [38] and ability to reduce tumor regression [39]” and the removal of references 37, 38, and 39;
- Table 2, Column “Outcome”, through the removal of “lower percentage area stenosis; improved neointimal proliferation and reduced tumor regression” and the addition of “more studies needed to be carried out for assessing the actual potential”.

With these corrections, the order of some references have been adjusted accordingly. The authors state that the scientific conclusions are unaffected. This correction was approved by the Academic Editor. The original publication has also been updated.
